# FAS-Based Cell Depletion Facilitates the Selective Isolation of Mouse Induced Pluripotent Stem Cells

**DOI:** 10.1371/journal.pone.0102171

**Published:** 2014-07-16

**Authors:** Eva Warlich, Axel Schambach, Dominik Lock, Dirk Wedekind, Silke Glage, Dominik Eckardt, Andreas Bosio, Sebastian Knöbel

**Affiliations:** 1 Miltenyi Biotec GmbH, Bergisch Gladbach, Germany; 2 Institute of Experimental Hematology, Hannover Medical School, Hannover, Germany; 3 REBIRTH Cluster of Excellence, Hannover, Germany; 4 Division of Hematology/Oncology, Boston Children's Hospital, Harvard Medical School, Boston, Massachusetts, United States of America; 5 Institute for Laboratory Animal Science, Hannover Medical School, Hannover, Germany; University of Freiburg, Germany

## Abstract

Cellular reprogramming of somatic cells into induced pluripotent stem cells (iPSC) opens up new avenues for basic research and regenerative medicine. However, the low efficiency of the procedure remains a major limitation. To identify iPSC, many studies to date relied on the activation of pluripotency-associated transcription factors. Such strategies are either retrospective or depend on genetically modified reporter cells. We aimed at identifying naturally occurring surface proteins in a systematic approach, focusing on antibody-targeted markers to enable live-cell identification and selective isolation. We tested 170 antibodies for differential expression between mouse embryonic fibroblasts (MEF) and mouse pluripotent stem cells (PSC). Differentially expressed markers were evaluated for their ability to identify and isolate iPSC in reprogramming cultures. Epithelial cell adhesion molecule (EPCAM) and stage-specific embryonic antigen 1 (SSEA1) were upregulated early during reprogramming and enabled enrichment of OCT4 expressing cells by magnetic cell sorting. Downregulation of somatic marker FAS was equally suitable to enrich OCT4 expressing cells, which has not been described so far. Furthermore, FAS downregulation correlated with viral transgene silencing. Finally, using the marker SSEA-1 we exemplified that magnetic separation enables the establishment of *bona fide* iPSC and propose strategies to enrich iPSC from a variety of human source tissues.

## Introduction

Pluripotent stem cells have long been considered a potent source for cell-based therapies. In 2006 Shinya Yamanaka's groundbreaking study paved the way to convert somatic cells into the so-called induced pluripotent stem cells (iPSC) [1], opening up new avenues for disease-specific drug modeling and patient-specific therapies. Rapidly, iPSC technology was proven to be a versatile tool for derivation of iPSC from healthy [2]; [3] and diseased [4]; [5] individuals and a proof-of-principle study demonstrated successful treatment of a genetic disorder via the iPSC interstage [6].

Reprogramming initiation was shown to be driven by a mesenchymal-to-epithelial transition, followed by a maturation phase before reaching a stably reprogrammed state [7]–[9]. An elaborate study investigating changes in mRNA and miRNA levels, histone modifications, and DNA methylation revealed that respective changes preferentially occur in two distinct waves [10]. An associated proteome analysis likewise observed bi-phasic expression changes and identified functional classes of proteins being differentially expressed in distinct phases [10]. Downregulation of fibroblast and mesenchymal markers was detected early in reprogramming and upregulation of epithelial markers shortly after [9]; [10]. Re-activation of several pluripotency-associated transcription factors (e.g. OCT4, *NANOG*, *SOX2*) is typically observed at intermediate or late stages of reprogramming displaying some degree of variability in the predictability of single markers for *bona fide* reprogrammed cells [10]–[14]. The first studies succeeding in induction of mouse iPSC took advantage of transgenic reporter systems linking reactivation of such pluripotency-associated gene promoters to either drug selection [1]; [15]–[17] or expression of fluorescent proteins [11]; [12] to identify the reprogrammed cells. While iPSC generated from a *Fbx15*-based reporter system failed to produce adult chimera, *Oct4*- and *Nanog*-based systems allowed the successful generation of germline-competent iPSC [1]; [15]–[17].

However, transgenic systems are labor-intense in their generation and cannot be employed when producing human iPSC for clinical purposes, rendering naturally expressed surface proteins an attractive alternative. Despite growing insight in gene expression changes in general and proteome changes in particular, a limited number of surface protein-based strategies have successfully been implemented that allow the discrimination of cellular subsets in reprogramming cultures. To date, no systematic investigation aiming at the identification of antibodies with the ability to discriminate reprogramming stages has been reported. MEFs undergoing reprogramming were shown to phenotypically progress from a THY1^+^ to a THY1^−^/SSEA1^−^ subpopulation, followed by a THY1^−^/SSEA1^+^ stage, ultimately achieving an SSEA1^+^/*Oct4*-GFP^+^ phenotype [10]; [12]. Accordingly, SSEA1 was successfully used to enrich for cells that had acquired pluripotency [10]–[12], as were EPCAM, E-CADHERIN [18] and combinations of PECAM1 with various other markers [19]. Likewise, a recent publication demonstrated the suitability of PGP-1 (CD44) and ICAM1 when combined with a Nanog-GFP-reporter [20]. SSEA1 and EPCAM were also successfully employed during directed differentiation depriving iPS-derived neuronal cells of remaining pluripotent cells of mouse and human origin, respectively [21]; [22].

In our study we sought to test a comprehensive library of 170 antibodies to identify surface proteins that are differentially expressed between MEF and PSC. The differentially expressed proteins should further be examined with regard to their dynamic expression changes in the course of reprogramming and their ability to enrich cells that are poised to become iPSC. Ultimately, we aimed to bypass low reprogramming efficiencies thereby easing the generation of iPSC lines.

## Results

### Twelve surface markers are differentially expressed between PSC and MEF

An antibody screening experiment was performed to identify surface markers that are expressed mutually exclusive on either mouse PSC or MEFs, thereby potentially allowing the discrimination of PSC in the heterogeneous cell mixture of reprogramming cultures. The screening was based on a library of 170 antibodies directed against mouse surface proteins (Table S1). The library consisted of antibodies that had been generated in house and commercially available antibodies, some of which were selected due to their potentially differential expression previously reported in the literature.

**Figure 1:**
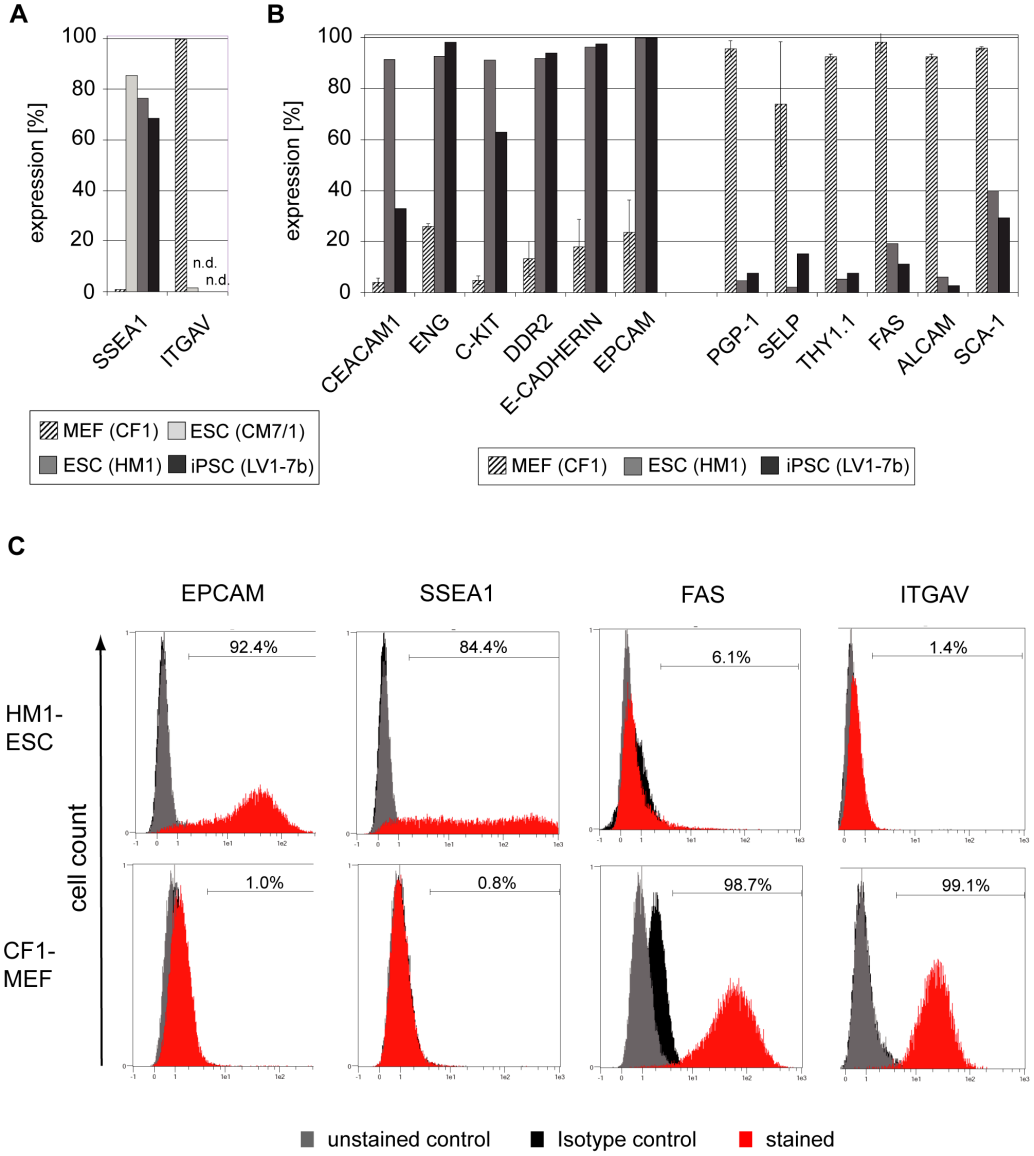
Identification of differentially expressed surface markers. A) A single flow cytometric analysis is shown to exemplify SSEA1 and ITGAV expression properties (n.d.  =  not determined), both of which were previously shown to be differentially expressed between MEF and PSC. B) Expression frequencies of antibody-targeted surface markers were tested by flow cytometry comparing MEFs (CF1), ESC line HM1 and iPSC line LV1-7b (n = 4 for MEFs: mean +/− SD; n = 2 for ESC/iPSC each). Given are the percentages of positive cells for identified candidate markers (6 potential pluripotency associated markers on the left-hand side and 6 potential MEF associated markers on the right-hand side). Expression data of all antibodies tested in the screen can be found in Table S1, additional expression characteristics on OG2-MEFs are shown in Figure S1. C) Representative histograms are shown for selected markers.

We compared expression by MEFs with the expression by mouse ESC and iPSC lines. Surface proteins were considered as potential reprogramming markers, if expression frequencies reached more than 90% on positive cell types and below 30% on the other cell types. Furthermore, markers were either designated as “MEF associated” or “pluripotency associated” markers with respect to their expression characteristics in our screening system. Beside the well described pluripotency associated marker SSEA1 and the MEF associated marker ITGAV (Fig. 1A and S.K., unpublished data) 12 candidate markers were identified. CEACAM1, ENG, C-KIT, DDR2, as well as the previously described proteins E-CADHERIN and EPCAM were identified as potential pluripotency associated markers. Six surface proteins were categorized as potential MEF associated markers (PGP-1, SELP, THY1.1, FAS, ALCAM and SCA-1) (Fig. 1B, C). Of note, the THY1 genotype is strain-dependent in mice. While CF1-MEFs expressed THY1.1, the *Oct4*-GFP (OG2)-MEFs (C57Bl/6J x C3H/HeN background) expressed THY1.2 (Fig. S1A). Though not meeting the aforementioned criteria, SELP was included for further evaluation because of a high standard deviation hampering interpretation. Interestingly, SELP was not expressed on OG2-MEFs, while no unexpected expression of the potential pluripotency associated markers was observed (Fig. S1A, B). We point out that a lack of protein detection in the given screen might also result from suboptimal antibody titers or specificity of the employed antibody clones. Hence, it might still be possible to detect some of the negatively tested proteins by different staining protocols.

In conclusion, we identified 12 potential reprogramming markers classifiable in MEF and pluripotency associated markers.

### Definition of reprogramming stages by activation and silencing of two combined reporter systems

**Figure 2:**
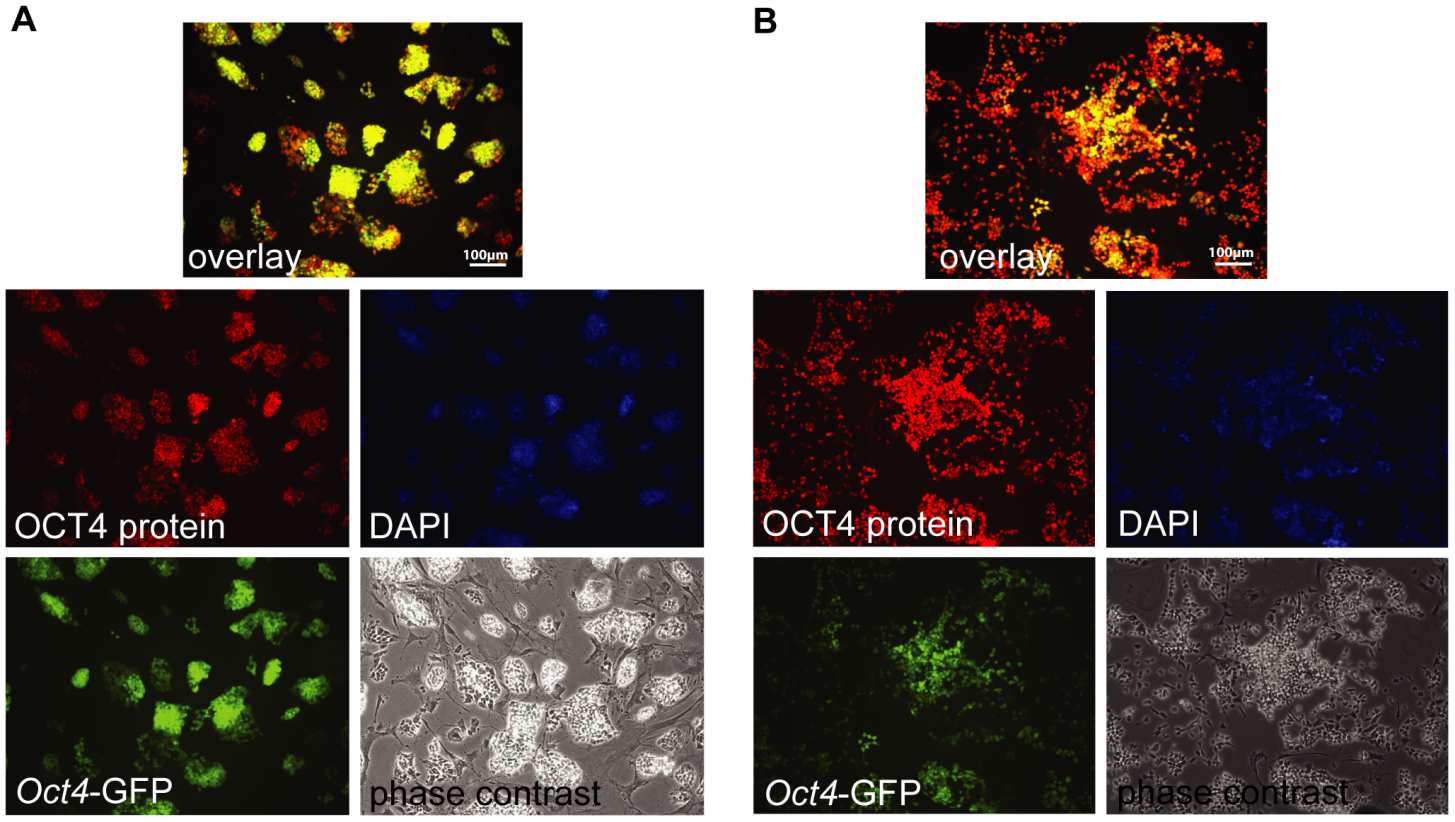
Oct4-GFP expression characteristics. A) Immunofluorescence of *Oct4*-GFP transgenic iPSC line LV1-7b cultured in non-differentiating conditions. Depicted are the *Oct4*-GFP marker, staining for OCT4 protein, a DAPI counterstain and phase contrast images. The overlay displays *Oct4*-GFP and OCT4 protein. B) The same iPSC line and analysis as in A cultured under differentiating conditions (2 day LIF withdrawal).

**Figure 3:**
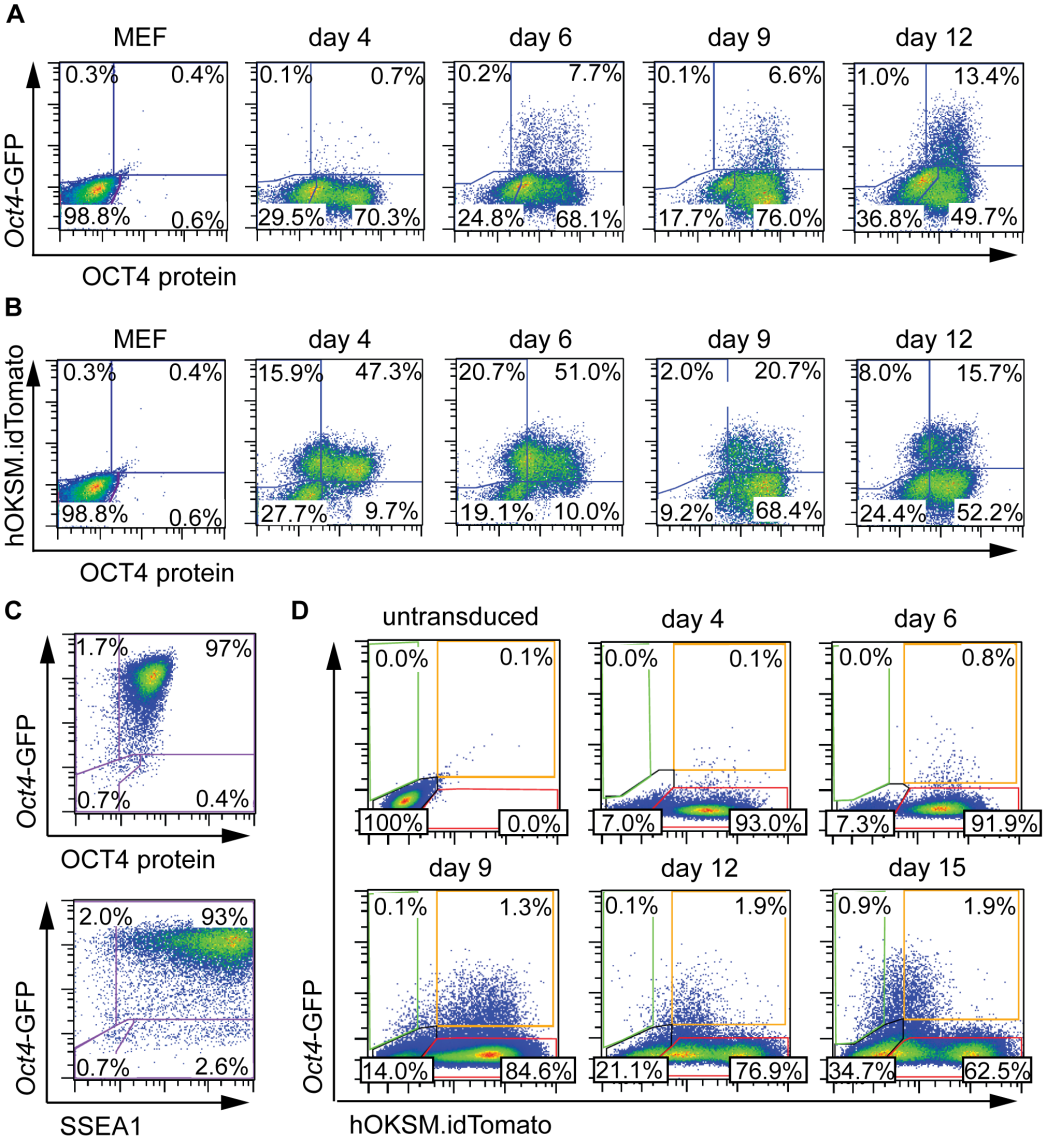
Definition of reprogramming stages by *Oct4*-GFP activation and transgene silencing. A, B) Correlation of OCT4 protein with the *Oct4*-GFP reporter expression (A) and the transgenic dTOMATO silencing (B), respectively, was investigated by flow cytometry in hOKSM.idTomato-transduced cells at different time points of reprogramming (day 4–12). Untransduced, unstained MEFs served as gating control. C) Expression characteristic of OCT4 protein, *Oct4*-GFP and SSEA1 as observed in established iPSC clones (passage 9). D) *Oct4*-GFP expression and transgenic dTOMATO silencing showed distinct expression characteristics in the course of reprogramming. dTOMATO expression was detectable early during reprogramming, followed by Tom^+^/GFP^+^ and Tom^−^/GFP^−^ intermediate stages, ultimately resulting in a Tom^−^/GFP^+^ fraction.

A reliable system to identify reprogrammed cells was needed before the expression characteristics of the candidate markers could be investigated in the reprogramming process. Therefore, a well-described *Oct4*-GFP pluripotency reporter mouse strain [23]–[25] was employed, that had previously been shown to be activated simultaneously or after silencing of lentiviral transgenes during reprogramming [26]. We observed an *Oct4*-GFP signal in most established iPSC that expressed OCT4 protein, but rarely in absence of OCT4 protein. However, in both standard culture (Fig. 2A) and differentiation-inducing conditions (Fig. 2B) many cells were observed in which OCT4 protein was detectable despite the absence of an *Oct4*-promoter dependent GFP signal. During reprogramming progression *Oct4*-GFP expressing cells exclusively arose as a subfraction of the OCT4 protein containing compartment (Fig. 3A). Flow cytometric analysis of established iPSC lines finally demonstrated co-expression of *Oct4*-GFP with OCT4 protein and SSEA1, respectively (Fig. 3C). Altogether, these observations indicate that live cell detection of *Oct4*-GFP likely underestimates the number of OCT4 expressing cells, most pronounced during early reprogramming, thus representing a very conservative marker of pluripotency induction in live cell imaging approaches.

Reprogramming was achieved by lentiviral transduction of h*Oct4*, h*Klf4*, h*Sox2* and h*c-Myc* (hOKSM), all co-expressed from a single transgenic construct in which reprogramming factor expression is linked by intergenic 2A peptides. In addition, a terminally IRES-linked coding sequence of dimeric *Tomato* (Tom) fluorescent protein enables tracking of reprogramming factor expression [26]. At early time points (day 4 p.t.) most of the OCT4 protein expressing cells co-expressed the dTOMATO reporter, while from day 9 p.t. the majority of OCT4-positive cells had silenced transgenes as indicated by loss of dTOMATO expression (Fig. 3D) suggesting reactivation of endogenous OCT4 synthesis.

Combining both reporter systems we found that dTOMATO was strongly expressed in transduced cells. First *Oct4*-GFP positive cells arose from this Tom^+^ fraction at day 4 p.t. (Fig. 3D). The mean fluorescence intensity pattern of dTOMATO altered over time discriminating a Tom^high^ and a Tom^low^ subpopulation which could clearly be distinguished from day 12 p.t. on. Importantly, the *Oct4*-GFP^+^ compartment was entirely Tom^low^ at that time point and subsequently further downregulated dTOMATO, indicating that this reporter combination represents a valuable tool to follow temporal reprogramming progression. Thus, a classification of the different reprogramming stages could be implemented that features (1) a Tom^+^ (single positive) early phase of reprogramming (2) a Tom^+^/GFP^+^ double positive intermediate phase and (3) an *Oct4*-GFP^+^ single positive late reprogramming stage. (4) Since from day 9 on far more cells expressed OCT4 protein as compared to *Oct4*-GFP or dTOMATO (Fig. 3A, B) an alternative intermediate phase (*Oct4*-GFP^−^/Tom^−^ double negative) was concluded, reflecting that *Oct4* promoter dependent GFP detection succeeded transcriptional activation of endogenous OCT4 expression. However, it is important to note that reprogramming cultures also contained non-transduced cells. Thus the *Oct4*-GFP^−^/Tom^−^ compartment consists of intermediate phase reprogrammed and untransduced cells. Due to a massive reduction of autofluorescence in the reprogrammed cell fraction as compared to the MEF population, the gating strategy for *Oct4*-GFP and dTOMATO expressing cells was performed rather strict, leaving out doubtful areas (total frequency of *Oct4*-GFP+ cells is 4.3% at day 15 p.t.).

Altogether, we were able to distinguish four distinct stages of reprogramming based on expression characteristics of reprogramming factors and *Oct4*-GFP reporter signals.

### EPCAM, SSEA1 and FAS expression changes reflect the reprogramming stages

**Figure 4:**
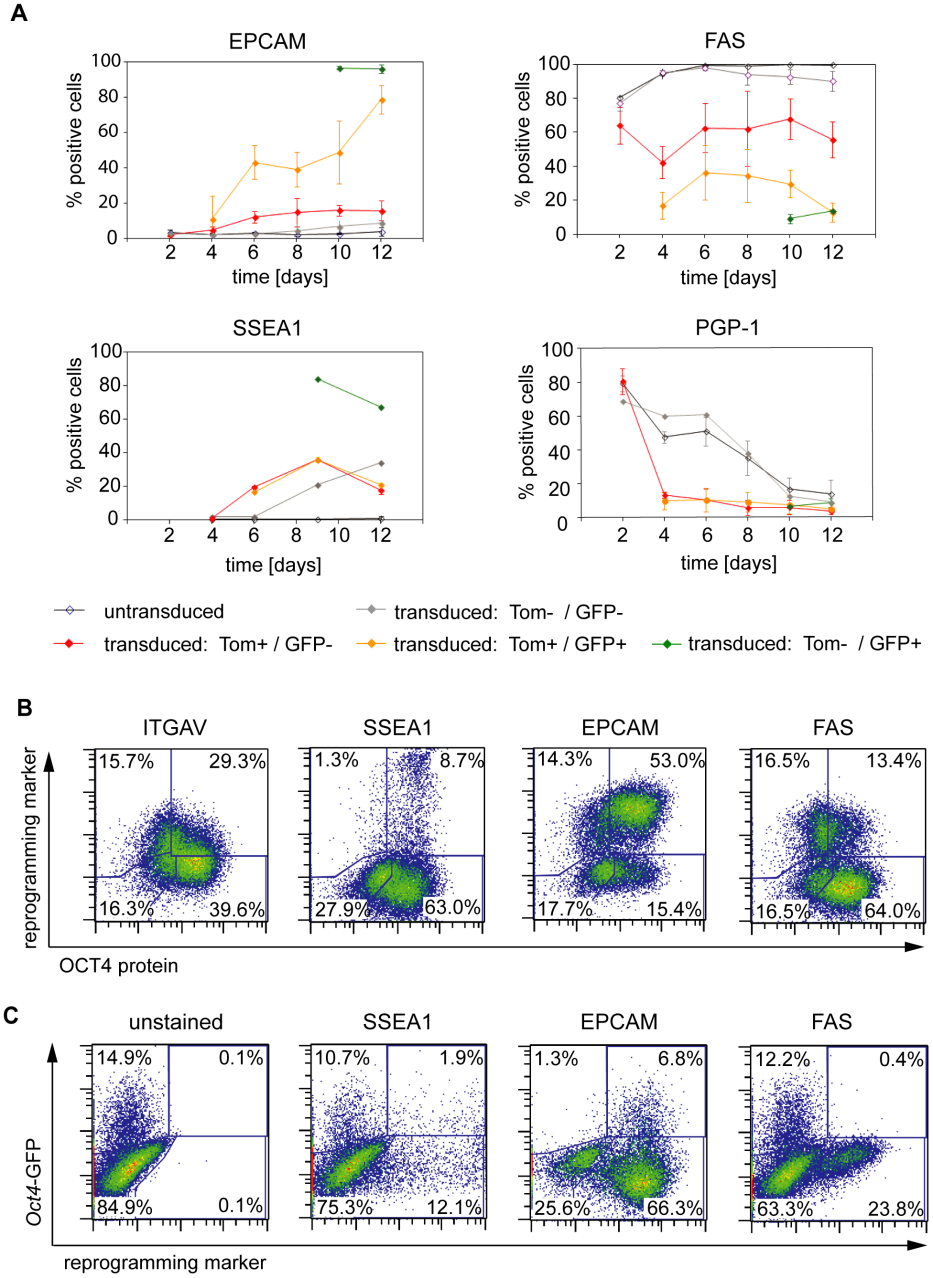
Expression kinetics of some candidate markers correlate with reprogramming stages. A) Expression frequencies (mean +/− SD) of candidate markers on reprogramming subpopulations were investigated by flow cytometry over time (n = 3: mean +/− SD; for SSEA1 n = 1) (also see Figure S2). Reprogramming subpopulations were defined as shown in Fig. 3D. B) Correlation of ITGAV, SSEA1, EPCAM and FAS with expression of OCT4 protein as analyzed by flow cytometry at day 12 p.t. C) Likewise, correlation of the selected candidate markers with the *Oct4*-GFP reporter system is shown at day 12 of reprogramming.

In order to investigate whether expression characteristics of the 12 candidate markers correlate with distinct stages of the reprogramming process, the expression of all markers was examined over time in the reprogramming stages defined above (Fig. 3D). We found that the potential pluripotency associated markers SSEA1 and EPCAM were gradually upregulated in the course of reprogramming leading to partial expression in the Tom^+^/GFP^+^ intermediate stage and high frequencies in the Tom^−^/GFP^+^ late stage (Fig. 4A). While CEACAM1 was upregulated only transiently, ENG failed to be upregulated at all (Fig. S2). C-KIT and DDR2 were only expressed by a marginal fraction of the *Oct4*-GFP^+^ cells.

MEF associated markers FAS and THY1.2 were highly expressed by untransduced OG2 cells, while striking downregulation could be observed in reprogramming stages as early as day 4 p.t., ultimately resulting in expression frequencies below 14% and 4% in the Tom^−^/GFP^+^ fraction, respectively (Fig. 4A and Fig. S2). PGP-1 downregulation occurred rapidly and, notably, not only in reprogramming but also untransduced cells lacking any clear correlation with the reprogramming status (Fig. 4A). SCA-1 downregulation was incomplete as implicated by a dramatic drop of the mean fluorescence intensity (not shown) while having little effect on the population frequency. ALCAM was not downregulated in any fraction or at any point of time (data not shown). ITGAV expression characteristics were difficult to interpret, because of a low expression dynamic of ITGAV in day 12 reprogramming cultures. Furthermore, negative correlation of ITGAV and OCT4 protein was incomplete (Fig. 4B). Consequently, ITGAV was neglected for subsequent experiments.

FAS downregulation occurred in all *Oct4*-GFP and most OCT4 protein expressing cells 12 days p.t., demonstrating a negative correlation between FAS and OCT4 protein (Fig. 4B, C). In contrast, the EPCAM^+^ subfraction predominantly correlated positively with expression of OCT4 protein. Although the SSEA1^+^ subfraction arose entirely from the cell population of OCT4 protein expressing cells, the majority of OCT4 protein expressing cells did not yet express SSEA1. This might indicate that detection of SSEA1 in our system lagged behind expression of OCT4 protein. Interestingly, the minority of *Oct4*-GFP expressing cells co-expressed SSEA1 and vice versa. This might indicate that SSEA1 and *Oct4*-GFP independently lag behind the expression of OCT4 protein (as detailed above).

We concluded that expression characteristics of SSEA1, EPCAM, FAS and THY1.2 are able to reflect reprogramming progression with EPCAM upregulation and FAS downregulation preceding the upregulation of SSEA1. We omitted ITGAV from further analysis due to its insufficient dynamic range in expression changes.

### Establishment of pluripotent stem cells lines from magnetically separated cells

**Figure 5:**
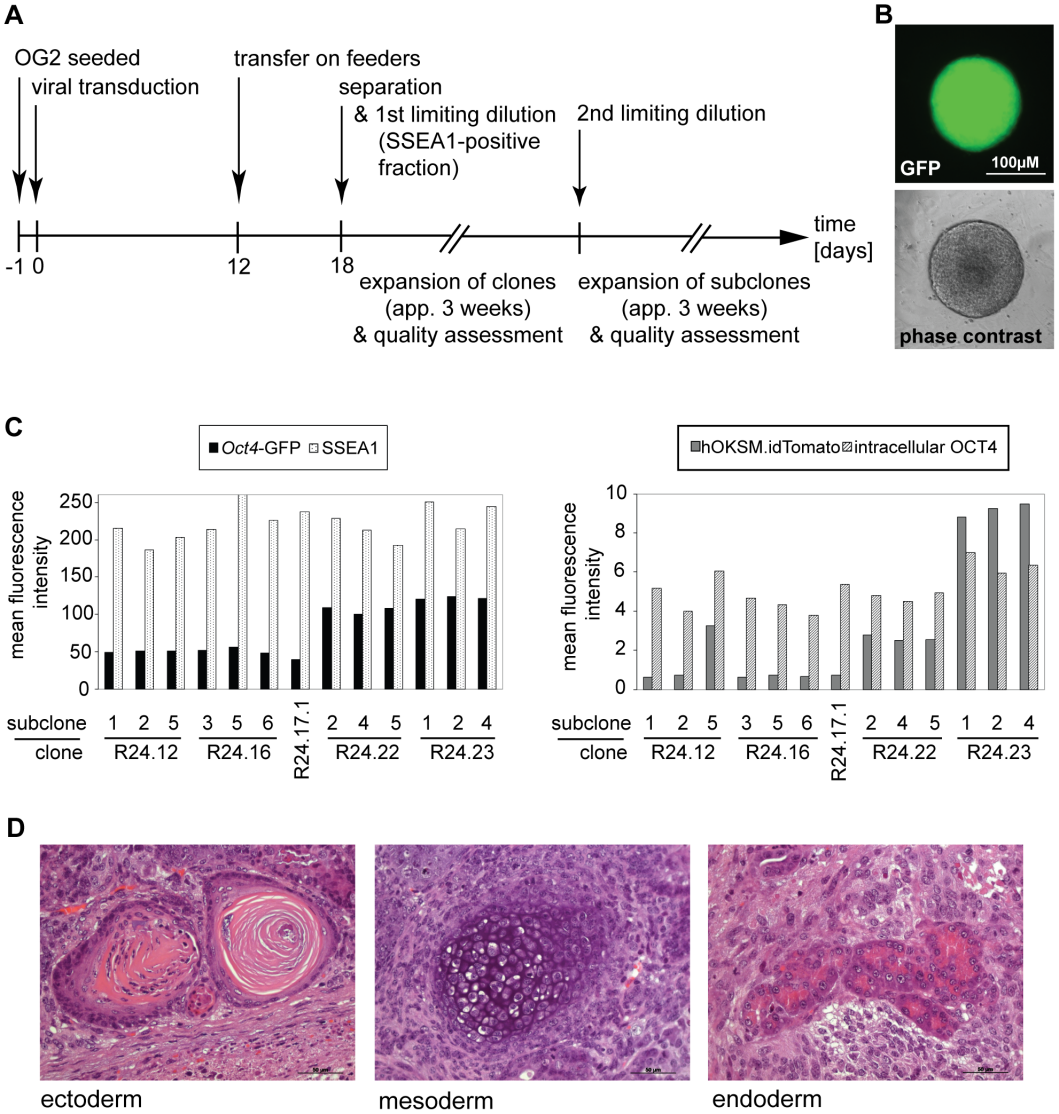
Establishment of iPSC lines from magnetically separated cells. A) Schematic overview of the procedure. After induction of reprogramming cells were once harvested and transferred on irradiated feeders. Isolation of SSEA1^+^ cells was performed 18 days p.t., diluted to a cell density of 5 cells/ml and 0.1 ml transferred per well of 96well plates (statistically 0.5 cells/well). B) 6–8 days after limiting dilution colonies of sufficient size and typical ESC-like morphology were chosen for expansion. Most colonies already demonstrated a strong and homogenous GFP signal. A representative colony is depicted. Expanded clones were subcloned in a second limiting dilution and expanded as described above. C) To pre-select subclones with the most promising potential for pluripotency several subclones of each clone were screened for expression levels of *Oct4*-GFP and SSEA1 (left plot) as well as expression of intracellular OCT4 and silencing of transgenic dTOMATO (right plot, n = 1). D) When injected into immunodeficient mice, 3 out of 4 subclones gave rise to teratomas with differentiation into derivatives of the 3 germ layers. Depicted is representative subclone R24.16.5 that formed keratinizing epithelium (ectoderm), cartilage (mesoderm) and pancreas-like glandular structures (endoderm).

In a proof-of-principal study we aimed to establish pluripotent stem cell lines from magnetically separated cells. We chose a positive selection strategy based on the well described marker SSEA1 to demonstrate that iPSC can be established from a particle-bearing cell fraction. Cells were therefore reprogrammed by lentiviral transduction 1 day after seeding, transferred onto feeder cells 12 days p.t. and separated at day 18 p.t. (Fig. 5A). After magnetic enrichment for SSEA1, cells were seeded in limiting dilution assays. Colonies that grew to sufficient size within 6 days and displayed ESC morphology were chosen for expansion (Fig. 5B). At this stage the clones did also exhibit a homogenous *Oct4*-GFP signal. Marker expression was quantified by flow cytometry demonstrating that all clones consisted of at least 75% SSEA1^+^ and >90% *Oct4*-GFP^+^ cells (data not shown). 6 clones were subcloned in a second round of limiting dilutions. Flow cytometric analysis was utilized to assess expression of SSEA1, *Oct4*-GFP, OCT4 protein, and remaining expression of transgenic dTOMATO in a screening experiment to preselect subclones for further investigation (Fig. 5C). Some subclones demonstrated residual dTOMATO expression (e.g. the derivatives of R24.23) and differences between the expression levels of the *Oct4*-GFP became obvious. All subclones expressed high levels of SSEA1 and demonstrated a robust expression of intracellular OCT4 protein. 4 subclones were chosen, each originating from a different parental clone, and tested in teratoma assays. 3 out of 4 subclones formed tumors consisting of differentiated tissues originating from the 3 germ layers (Fig. 5D). Importantly, the subclone (R24.23.4) that failed to produce a tumor exhibited residual transgene expression at high levels.

In line with previous reports [27], we demonstrated that the procedure of magnetic cell sorting is well suited for establishment of pluripotent stem cell lines.

### Separation of SSEA1^+^, EPCAM^+^ or FAS^−^ enriches cells committed to become iPSC

**Figure 6:**
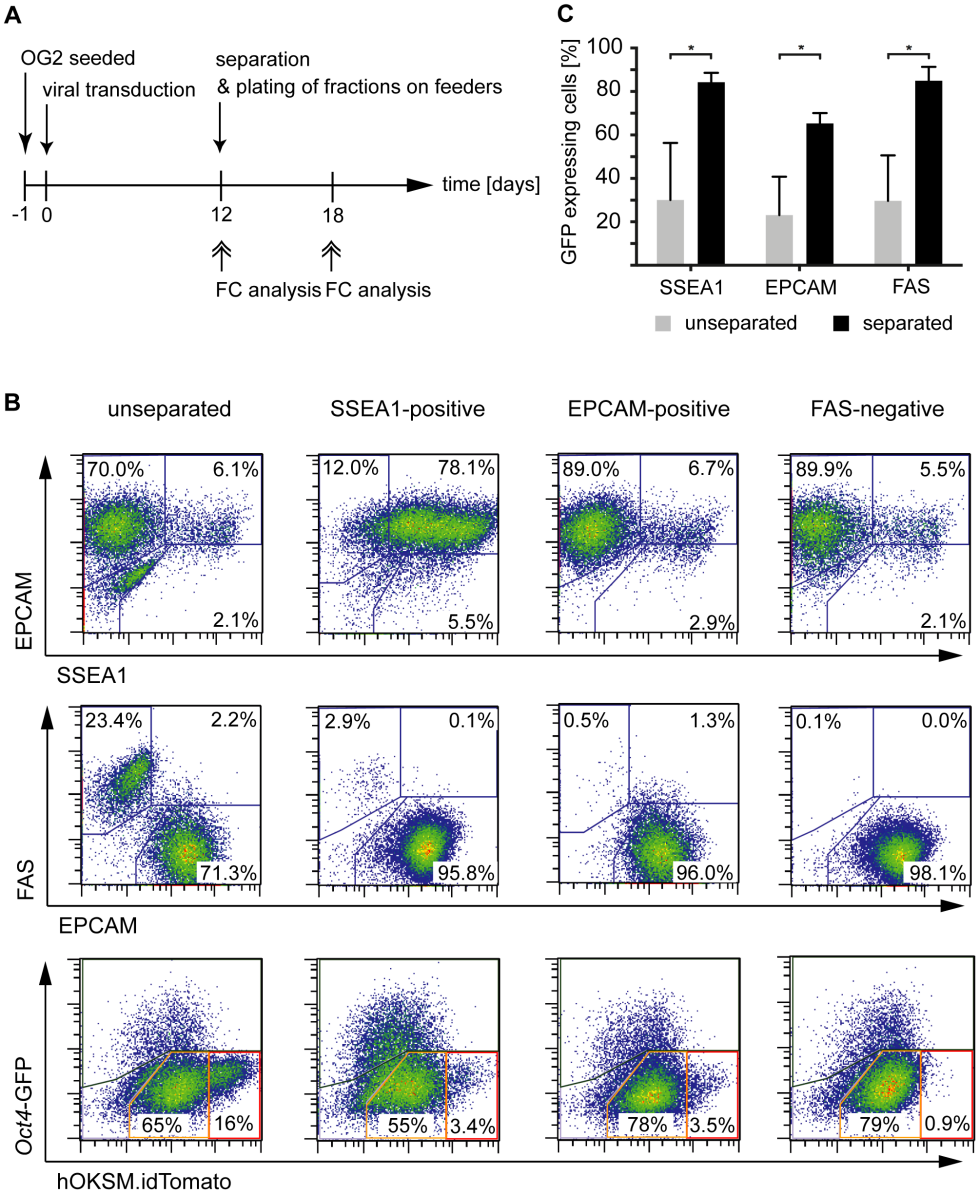
Separation of SSEA1^+^, EPCAM^+^ or FAS^−^ cells allows for enrichment of *Oct4*-GFP reporter-positive cells. A) Timeline of reprogramming, cell separation and analyses. OG2 were seeded one day prior to viral transduction. Cells were magnetically separated at day 12 p.t. (see Figure S3 for efficiencies) and fractions further cultured on irradiated feeder cells for another 6 days. Flow cytometric analyses of all separated fractions were performed directly after separation and after further culture. B) Depicted are the expression characteristics of the target fractions of each separation strategy (SSEA1^+^, EPCAM^+^ and FAS^−^) as measured directly after separation by flow cytometry. Pairwise correlations of SSEA1/EPCAM (upper panel), FAS/EPCAM (middle panel) and hOKSM.idTomato/*Oct4*-GFP (lower panel) are shown. Unseparated cells are shown in the left column. C) The percentages of *Oct4*-GFP expressing cells in the different fractions are shown for magnetic separations based on SSEA1, EPCAM or FAS. Analyses were performed 6 days after separation by flow cytometry. p values were calculated with a Student's t test. *p<0.05; **p<0.01; ***p<0.001. Scale bars show the SD for three separate experiments (alpha = 95%).

We next sought to examine whether separation by alternative markers, i.e. the identified reprogramming markers EPCAM and FAS, is able to improve the procedure in terms of cell yield or phenotype of the target fraction. Twelve days after induction of reprogramming in OG2-MEFs magnetic separations were performed comparing the enrichment of SSEA1^+^ or EPCAM^+^ and the depletion of FAS^+^ cells (Fig. 6A). A representative example for the efficiency of each separation strategy is given in Figure S3. The desired target fractions (SSEA1^+^, EPCAM^+^, FAS^−^) typically achieved purities of 89%, 98% and 98%, respectively (Fig S3). Each fraction was seeded on top of feeder cells and subsequently cultured for another 6 days.

Flow cytometric analysis directly after separation demonstrated that the phenotype of any target fraction was entirely composed of EPCAM^+^/FAS^−^ cells independent of the administered separation strategy (Fig. 6B). Importantly, the SSEA1^+^ cells represented a subfraction of the EPCAM^+^ (FAS^−^) cells. Consequently, SSEA1 enrichment yielded 6-fold less cells (data not shown), but led to an accordingly higher population frequency of SSEA1^+^ cells. Remarkably, only FAS depletion completely eliminated the Tom^high^ subpopulation in the target fraction, thereby removing cells with diminished transgene silencing.

Six days after separation all three separation strategies (FAS, SSEA1 and EPCAM) had led to a similar and significant enrichment of the *Oct4*-GFP expressing cells (85%, 84% and 65%, respectively), confirming that any of the given strategies is suitable to enrich cells poised to become iPSC (Fig. 6C). Notably, though the respective magnetic isolation protocols were mainly optimized to yield highly pure target fractions (i.e. the column flow-through or “negative” fraction for FAS, and eluted or “positive” fraction for SSEA-1 and EPCAM, Fig S3), reduced ratios of GFP+ cells (yet statistically insignificant) were observed in the non-target fractions of FAS and EPCAM when compared to the unseparated cells 6 days after separation (data not shown).

In summary, both EPCAM enrichment and FAS depletion were characterized by an enhanced cell yield compared to SSEA1 enrichment. FAS depletion represented the only strategy that enabled the complete removal of the transgene expressing Tom^high^ cell population.

### Tissue distribution of potential reprogramming markers on various human cell types

**Figure 7:**
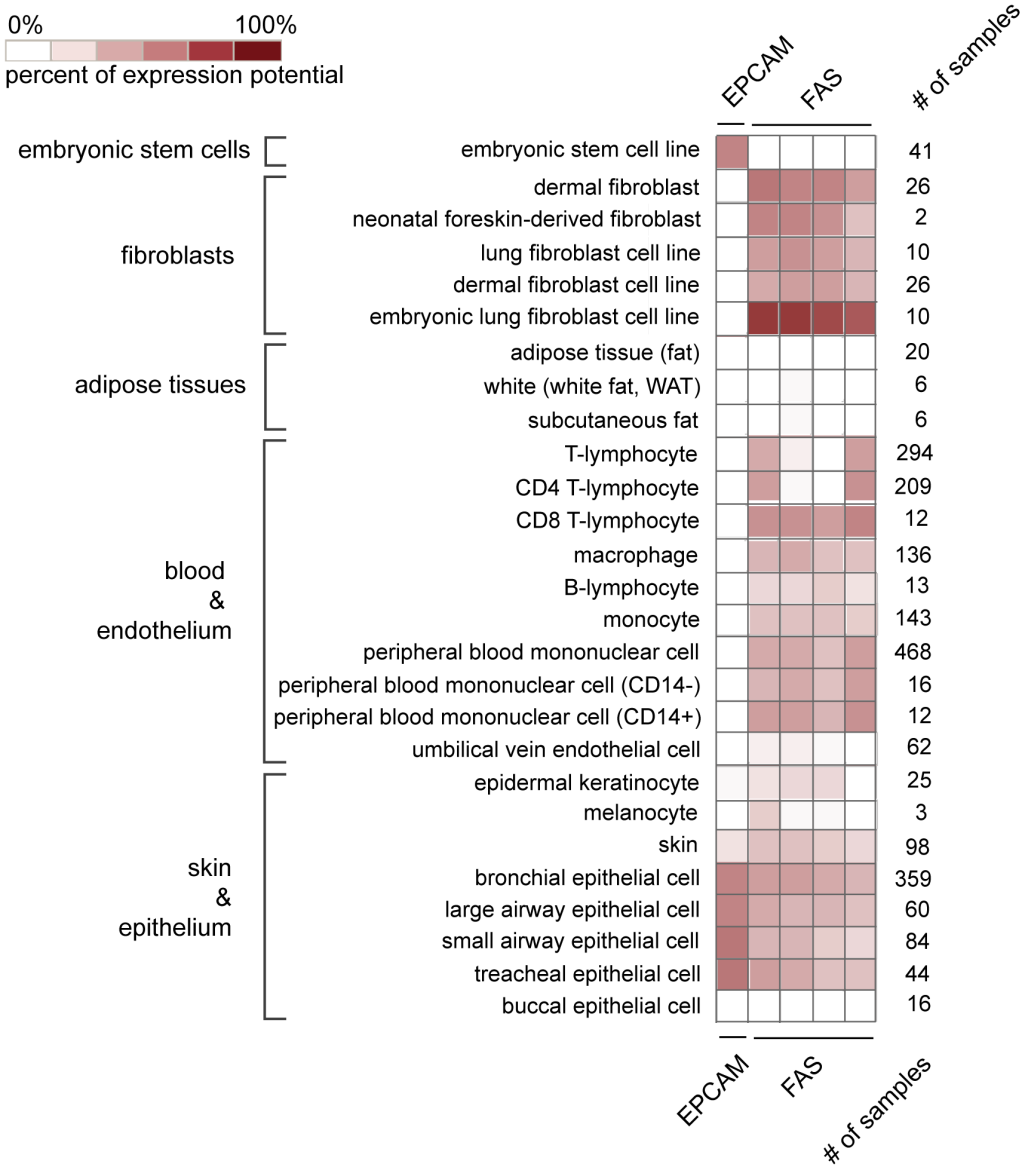
Excerpt of the tissue distribution of EPCAM and FAS mRNA in human cell types. An mRNA analysis based on the Genevestigator software tool was performed reflecting the tissue distribution of investigated mRNAs. Shown are selected tissues that are relatively easily accessible and have already been reprogrammed in previous reports. The “percentage of expression potential” represents the average expression of a gene across all samples of the particular cell type as compared to the sample with the highest expression (maximum level, 100%) for the particular gene [percentage of expression potential  =  average/maximum]. The number of samples that were included to calculate this average is indicated on the right side of the graph. Results are given as logarithmic (log2) heat map.

**Figure 8:**
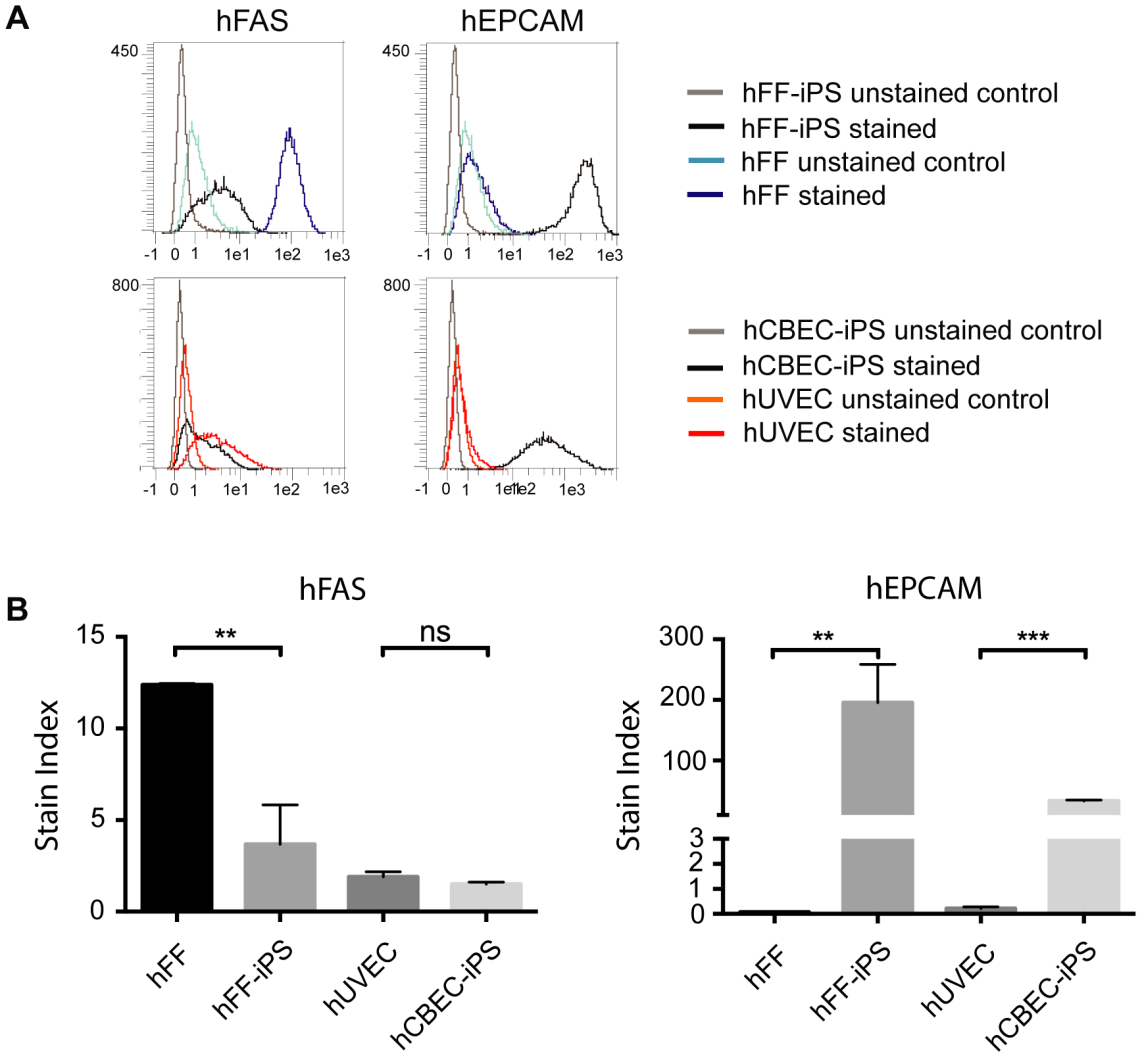
Expression analysis of EPCAM and FAS protein in selected human cell types. A, B) Flow cytometry analysis of FAS and EPCAM expression on human foreskin fibroblasts (hFF), human umbilical vein endothelial cells (hUVEC) and two hiPSC lines. A) Representative flow cytometry analysis results. B,) Independent quantification of relative expression levels of FAS and EPCAM for the same four cell types. Stain indices (SI) are depicted to enable the comparison of expression levels for cell types displaying different levels of autofluorescence. SI were calculated as follows: (Median of labeled cells – Median of unlabeled cells)/(2× standard deviation of unlabeled cells). p values were calculated with a Student's t test. *p<0.05; **p<0.01; ***p<0.001. Scale bars show the SD for three separate experiments (alpha = 95%).

The tissue distribution of some of the candidate markers on various human cell types was investigated using the Genevestigator software tool [28], which utilizes publicly available microarray data sets. To estimate whether the markers might also be suitable to selectively enrich reprogrammed cells from alternative human source tissues, we examined mRNA expression of the candidate genes in different tissues and cell lines. The excerpt given in Figure 7 focuses on cell types that are easily accessible and had repeatedly been reprogrammed in previous reports (fibroblasts, blood cells, skin, adipocytes). mRNA of pluripotency associated marker EPCAM had not been detected in human fibroblasts, adipose tissue and various blood cell subtypes (Fig. 7). In line with these data we found EPCAM protein to be expressed by hiPSC lines, but not human foreskin fibroblasts (hFF) and human umbilical vein endothelial cells (hUVEC) (Fig. 8A, B). It might thus be possible to also employ EPCAM-enrichment for isolation of iPSC in the human system, if fibroblasts or endothelial cells are to be reprogrammed. Since EPCAM is a well known epithelial surface protein, its mRNA was expressed in epithelial cell sources (Fig. 7), arguing against hiPSC enrichment via EPCAM from this cell type. In contrast, mRNA of somatic marker FAS seemed to be expressed not only by fibroblasts and blood cells but also epithelial cells (Fig. 7), thereby potentially enabling the isolation of hiPSC from epithelial tissues. While FAS protein was indeed found to be expressed significantly less in hiPSC than in hFF (Fig. 8A, B), a subpopulation of these hiPSC expressed low amounts of FAS protein. Noteworthy, FAS protein was only weakly expressed in hUVEC. Future investigations are therefore required to assess the suitability of FAS (and also EPCAM) for isolation of iPSC from human tissues.

Altogether, we demonstrated the suitability of SSEA1, EPCAM and FAS for enrichment of iPSC from mouse embryonic fibroblasts. Moreover, we hypothesize that EPCAM and FAS selection strategies might also be useful for isolation of iPSC from various human cell types.

## Discussion

Usage of surface markers to identify reprogramming stages can be readily applied to unmodified cells and allows for live-cell imaging and antibody-based separation strategies. Accordingly, some differentially regulated mouse surface markers have already been utilized for selective isolation of reprogramming subpopulations. These markers include SSEA1, which was previously shown to be upregulated as one of the earliest markers in reprogramming [11]; [12]. EPCAM, which has been shown to actively promote cellular reprogramming [29], also allowed for successful enrichment of NANOG-expressing cells [18]. Combinations of PECAM1 with SSEA1, ITGA6, PVRL2 and EPCAM, respectively, were shown to enrich the fraction of pluripotency factor expressing cells [19]. Also, the combination of PGP-1/ICAM1/Nanog-GFP was recently suggested to provide high-resolution information during late pluripotency gene upregulation [20], although without providing a generic marker code enabling the isolation of iPSC generated from wild type cells. Likewise though with different intention surface marker SSEA1 was used to deprive iPS-derived neuronal cells of remaining pluripotent cells [22] Nonetheless, only limited numbers of antibodies had so far been tested for their potential to discriminate distinct stages of reprogramming.

We therefore aimed to employ a panel of 170 antibodies to detect expression of surface proteins on MEFs and PSC and were able to identify 12 differentially expressed proteins. We investigated expression changes of these respective markers in distinct reprogramming subpopulations. Combining a traceable lentiviral expression system with a transgenic *Oct4*-GFP reporter system, we were able to define four distinct reprogramming stages. These stages were characterized by silencing of vector transgenes, which was previously described as hallmark of reprogramming [12]; [15]–[17]; [26], and reactivation of the *Oct4*-GFP reporter. Previous reports demonstrated the suitability of combined approaches of transgene silencing and pluripotency factor (NANOG, SOX2) reactivation that were shown to reflect progression through the different stages of reprogramming [14]; [30]. We found that reporter signals from our transgenic *Oct4*-GFP cassette succeeded the endogenous reactivation of OCT4 protein during reprogramming but established a full correlation in established iPSC, representing a conservative indicator of OCT4 expression. Despite the delayed expression characteristics of the *Oct4*-GFP signal, it correlated entirely with the expression intensity of the reprogramming factors, i.e. first with the Tom^low^ and later with the Tom^−^ subpopulation. We concluded that our combined system is thus suitable to define sequential reprogramming stages.

Employing this two-reporter-system we were able to investigate the kinetic changes of our potential reprogramming markers in the course of reprogramming. We confirmed the sequential upregulation of first EPCAM and second SSEA1 [10]–[12]; [31]. Previous studies had revealed a function of EPCAM in maintenance of the undifferentiated phenotype in mouse and human ESC [32]; [33]. Importantly, this function is exerted by regulation of pluripotency-associated factors, such as OCT4, SOX2, KLF4, C-MYC and NANOG [33]. In line with these findings, we could observe that EPCAM expression correlated with expression of OCT4 protein even in cells that had already silenced the transgenic reprogramming factors as indicated by loss of Tom expression. Noteworthy, a recent study suggested EPCAM to be as informative to predict the iPSC state as NANOG and LIN28 [31]. Since upregulation of EPCAM precedes the expression of SSEA1, the latter marker was shown to detect only a subpopulation of the OCT4 expressing (EPCAM^+^) cells. Interestingly, besides SSEA1 also the *Oct4*-GFP reporter succeeded OCT4 protein expression. However, both markers did not correlate with one another in early reprogramming, suggesting independent induction of the two markers. Co-expression with robust expression levels was finally observed after establishment of iPSC lines. While our antibody screen had revealed expression of C-KIT by PSC, we observed only marginal expression in the *Oct4*-GFP single positive stage until day 12 of reprogramming. Recent data suggest that this protein might represent an intermediate marker of reprogramming progression [31] and would thus also be an interesting candidate for separation of iPSC. Likewise, DDR2, which demonstrated similar expression kinetics as C-KIT in our study, might be an additional candidate for further experiments. Interestingly, our data does not corroborate the recently reported ICAM1+/PGP-1- signature of late reprogramming stages [20]. We found ICAM1 to be broadly expressed on the MEF population as well as on iPS (Table S1). PGP-1 was excluded from isolation studies due to the lacking correlation with mature reprogramming stages defined by our dual reporter system.

To our knowledge, we are the first to characterize expression dynamics of FAS in the course of reprogramming of MEF into iPSC. FAS was quickly downregulated on OG2-MEFs upon induction of reprogramming. This is well in accordance with previous reports suggesting the absence of FAS expression in naïve mouse ES cells [34]; [35]. Furthermore, the expression of FAS was reported to be regulated by cooperating transcription factors, e.g. repression of FAS expression by OCT4 [36]; [37]. This is in line with our data demonstrating anti-correlation of FAS and OCT4 protein expression. In addition, activated tumor suppressor protein P53 positively correlates with FAS expression levels [37]–[40] and vice versa. Importantly, inhibition of P53 has been shown to enhance reprogramming efficiencies [41]. It would thus be interesting to investigate, whether FAS downregulation actively promotes reprogramming or is a consequence of negative regulation by OCT4 and lacking upregulation by P53.

In our study we were able to confirm, that not only pluripotency marker upregulation but also downregulation of a somatic marker can be applied when isolating iPSC [20]. We suggest that downregulation of FAS, which is strongly expressed on MEFs, enables the enrichment of cells poised to become iPSC. A direct comparison of EPCAM- or SSEA1-enrichment with FAS-depletion demonstrated similar frequencies of *Oct4*-GFP^+^ cells in the SSEA1^+^ and FAS^−^ target fractions after a subsequent culture period. Though all sorting paradigms led to a robust enrichment of *Oct4*-GFP^+^ cells, minor fractions of *Oct4*-GFP^−^ were detectable 6d after isolation. These *Oct4*-GFP^−^ could represent either a) spontaneously differentiated iPSC, b) reprogramming cells with retarded kinetics or c) cells which have not fully matured after having initiated the reprogramming process [42]. Isolation of the EPCAM^+^ fraction resulted in a slightly lower frequency of *Oct4*-GFP^+^ cells. This is in line with our observation that SSEA1^+^ cells arose as a subfraction of the EPCAM^+^ population, reflecting that EPCAM is upregulated even before SSEA1. Although the SSEA1^+^ cells also represent a subpopulation of FAS^−^ cells, FAS depletion worked equally well as SSEA1-enrichment. In consequence, selection strategies based on EPCAM as well as FAS yield higher cell numbers with comparable potential to SSEA1^+^ cells. Considering that only depletion of FAS expressing cells was able to completely abolish the fraction of immature, transgene dependent Tom^high^ cells from the reprogramming culture, it is tempting to hypothesize that lack of FAS indicates a more mature reprogramming stage with a higher degree of epigenetic remodeling. It would thus be interesting to address the functional relevance of FAS for cellular reprogramming in future studies.

By enrichment of SSEA1^+^ cells we have exemplified that the procedure of magnetic separation is well suited for the establishment of pluripotent stem cell lines even from a particle-bearing cell fraction. Established iPSC lines demonstrated pluripotency to the level of teratoma formation with differentiation into tissues derived from the 3 germ layers. Likewise, Dick and colleagues observed no adverse effects of magnetic particles when transducing cells with magnetic-particle-bearing lentiviruses and were able to establish hiPSC lines from positively selected human cells [27]. Importantly, magnetically separated cells are frequently reported to result in stable engraftment and survival of transplanted cells *in vivo*, e.g. using ES-derived cells [43]; [44] or in cancer treatment [45]–[49]. Thus, magnetic separation is suitable for rapid enrichment of pluripotent cells and a robust method for clinical therapies.

To date, we demonstrated the selective enrichment of iPSC derived from mouse embryonic fibroblasts. However, for future clinical applications selection strategies for human iPSC are needed. In addition, it might be of interest to reprogram cell types that are more easily accessible and can be obtained in sufficient amounts. We thus inspected the mRNA expression patterns as observed in various human tissues based on numerous publicly available microarray data sets. These data suggest that FAS might serve to isolate hiPSC from epithelial cells, while EPCAM as known epithelial marker cannot be employed. This example highlights the need to take the cellular background into account when selecting a suitable marker for separation. Protein data moreover demonstrated significant expression differences of FAS between fibroblasts and hiPSC derived thereof. Furthermore, EPCAM expression differed significantly between hFF and hFF-derived iPSC and hUVEC and hCBEC-derived iPSC, respectively. Although the collective data support the notion that FAS and EPCAM might also be suitable to isolate iPSC in the human system, expression kinetics need to be investigated and separations to be tested to draw definite conclusions. Noteworthy, in contrast to its expression on mouse pluripotent stem cells SSEA1 is only expressed on a differentiating subpopulation of human PSC cultures [50]; [51]. Therefore, it cannot be used to positively select for potential iPSC from human reprogramming cultures. Instead, alternative markers have been employed in proof-of-principle studies including SSEA4, TRA-1-60, TRA-1-81 and TNSFS8 (CD30) [27]; [52]–[56]. Additionally, combinations of the positive markers SSEA4 and TRA-1-60 with the negative marker Aminopeptidase N (CD13) have been used for selection of human pluripotent stem cells [56].

In conclusion, we reported 12 naturally occurring surface proteins that are differentially expressed between mouse embryonic fibroblasts and pluripotent stem cells. SSEA1, EPCAM and FAS allow the selective enrichment of cells poised to become iPSC from reprogramming MEF cultures by magnetic separation, thereby overcoming low efficiencies and easing the generation of iPSC lines. We hypothesize that some of these separation strategies can also be used to enrich iPSC from human source tissues and that they can aid the generation of patient- and disease-specific iPSC for research and clinical applications.

## Materials and Methods

### Ethical Statement

This study was conducted in accordance with the German animal protection law and with the European Communities Council Directive 86/609/EEC for the protection of animals used for experimental purposes. All experiments were approved by the Hannover Medical School Institutional Animal Care and Research Advisory Committee and permitted by the local government (LAVES, permit number 10/0209) according with the German animal protection law and with the European Communities Council Directive 86/609/EEC for the protection of animals used for experimental purposes.

### Cell culture

293T human epithelial kidney cells (CCL-121) and HT1080 human fibrosarcoma cells (CRL-3216) were purchased from ATCC and maintained in Dulbecco's modified Eagle's medium (Miltenyi Biotec, Bergisch Gladbach, Germany), 10% fetal calf serum (FCS) (PAA, Cölbe, Germany). MEFs were prepared from day 13.5 embryos of CF1 (Charles River) or *Oct4*-GFP (OG2) mice strains [25] and cultured on 0.1% gelatin-coated dishes in MEF medium (Dulbecco's modified Eagle's medium, 10% FCS, 1% non-essential amino acids (NEAA), 2 mM L-glutamine (all from PAA) and 100 µM β-mercaptoethanol (β-ME) (Sigma-Aldrich, Munich, Germany)). Feeder-dependent mouse ES cells (HM1) [57], obtained from Dr. Douglas Melton (Harvard University Cambridge, MA, USA), the iPS-line LV1-7b [24] and newly generated iPS cells were cultured on gamma-irradiated CF1-MEFs in iPSC medium (Knockout-DMEM (Invitrogen, Darmstadt, Germany), 15% ES-grade FCS (PAN, Aidenbach, Germany), 1% NEAA, 2 mM L-glutamine, 100 µM β-ME and 22 U/ml mouse LIF (Miltenyi Biotec). Mouse PSC lines were splitted every two days seeding 1–2×10^5^ cells per 6 well dish. Optionally, mouse PSC were additionally cultured under “3i” conditions [58]–[60] (1 µM PD0325901, 3 µM CHIR99021 and 4 µM SB431542; all from Stemgent, Cambridge, MA, USA). Feeder-independent mouse ES cells (CM7/1) [61], obtained from Dr. Robert Zweigerdt (REBIRTH, Hannover Medical School, Germany) were cultured on 0.1% gelatin-coated plates in ES-medium (DMEM, 10% ES-grade FCS, 1% NEAA and 100 µM β-ME and 22 U/ml mouse LIF). Human BJ-fibroblast (08-0027) were purchased from Stemgent and maintained in Dulbecco's modified Eagle's medium supplied with 10% FCS, 1% non-essential amino acids (NEAA), 2 mM L-glutamine (all from PAA). Human BJ-fibroblast-derived iPSC (hFF-iPS) were generated using the mRNA Reprogramming Kit (Stemgent) according to the manufacturer's instructions. Human cord blood endothelial cell-derived iPSC (hCBEC-iPS) [62], obtained from Dr. Ulrich Martin (REBIRTH, Hannover Medical School, Germany) and hFF-iPS were maintained on gamma-irradiated CF1-MEFs in hiPSC medium (D-MEM/F12, 20% KO-Serum Replacement (Invitrogen), 1% NEAA, 1 mM L-glutamine, 100 µM β-ME, 8 ng/ml human FGF-2 premium grade (Miltenyi Biotec)). Cells were passaged using TrypLE Select (Invitrogen). Medium was supplemented with Cloning & Recovery Supplement (Stemgent) for the first 2 days after replating.

### Virus production

Lentiviral particle production was performed as described previously [63]. A 4-in-1 reprogramming vector harboring 2A-linked h*Oct4*, h*Klf4*, h*Sox2* and h*c-Myc* and an IRES-linked *dTomato* (hOKSM.idTomato) was used [26]. To determine biological titers, human HT1080 fibroblasts were transduced with viral supernatants and expression of virally delivered fluorescent protein dTOMATO was measured by flow cytometry 4 days post transduction (p.t.). Titers were calculated as follows: [(cell number at transduction) x (frequency of transduced cells) x 2]/(volume of viral supernatant). Viral transductions were performed in presence of 10 mM HEPES and 4 µg/ml protamine sulphate (Sigma) for 8–16 h.

### Flow cytometry

For the screening assay cells were harvested using 0.25% trypsin-EDTA. Reprogramming cultures were harvested as detailed in the “Reprogramming” paragraph of the Material and Methods section. For surface marker stains, primary antibody staining was performed in PEB buffer (PBS/2 mM EDTA/0.5% BSA) for 10 min at 4°C, if not stated otherwise. Antibodies and staining conditions of the antibody screening are listed in Table S1. Moreover, anti-mSSEA1, anti-mITGAV, anti-hCD95 and anti-hEPCAM were used according to manufacturer's instructions (all Miltenyi Biotec). Cells were washed once and, if required, secondary staining also performed for 10 min at 4°C. Virally transduced cells were additionally fixed in 1.85% formaldehyde (Miltenyi Biotec) for 20 min at room temperature before flow cytometric analysis.

Staining for intracellular OCT4 was conducted after surface marker staining. According to manufacturer's instructions (BD, Heidelberg, Germany) cells were fixed in a 1∶1 mixture of Cytofix and Cytoperm for 20 min at 4°C and subsequently washed in Perm/Wash solution. The OCT4 intracellular stain was conducted using anti-Oct4 Alexa Fluor 647 (BD, Heidelberg, Germany) for 30 min at 4°C and cells were again washed in Perm/Wash. For flow cytometric analysis cells were resuspended in PEB buffer. Data were acquired using the MACSQuant Analyzer or MACSQuant VYB and analyzed with the MACSQuantify Software.

Stain indices (SI) were calculated as follows: (Median of labeled cells – Median of unlabeled cells)/(2× standard deviation of unlabeled cells).

### Immunofluorescence

Cells grown in standard culture dishes were rinsed with PBS, fixed with 4% paraformaldehyde (Merck), permeabilized with 0.1% Triton X-100 (Sigma-Aldrich) and blocked with 10% FCS in PBS. Cells were incubated with Anti Oct-4 antibody (Santa Cruz (Heidelberg, Germany) sc-5279, 1∶50) in blocking solution for 1 h at 4°C. Secondary antibody staining was performed for 45 min (goat anti-mouse-Alexa Fluor 594, Invitrogen), followed by DAPI staining (Sigma) for 5 min. Cells were covered by mounting medium (Invitrogen) and analyzed using a Nikon Eclipse TS 100.

### Reprogramming

For reprogramming 6,5×10^3^ MEFs per cm^2^ were seeded on gelatin-coated dishes one day prior to transduction. MEFs were transduced virally using a multiplicity of infection of 4–7. Medium was changed to MEF medium 8–16 h p.t. Cells were further cultured in MEF medium until day 4 p.t. and in iPSC medium hereafter. Medium was exchanged every other day supplemented with 2 mM valproic acid (Merck, Darmstadt, Germany) from day 2 onwards, 25 µg/ml vitamin C (Sigma-Aldrich) from day 2–8 and “3i” cocktail from day 8 onwards. To harvest the cells, dishes were washed once in PBS, pre-treated with 1 mg/ml Dispase (Roche, Penzberg, Germany) for 7 min at 37°C, washed and dissociated in 0.25% trypsin-EDTA (Sigma-Aldrich) for 5 min at 37°C.

### Magnetic cell separation

Reprogrammed cells were harvested as described above. Cell suspensions were filtered via 30 µM pre-separation filters (Miltenyi Biotec). Magnetic separations were performed according to manufacturer's protocol. In brief, 5×10^6^ cells were magnetically labeled as follows. For FAS separation (indirect separation) cells were first stained with 1 µg/ml primary antibody (Anti-FAS-Biotin) for 10 min at 4°C in 0.1 ml of PEB buffer, washed by addition of 2 ml buffer, centrifuged and resuspended in buffer. Magnetic labeling in general was performed for 15 min at 4°C in 0.1 ml of a 1∶5 dilution of the according MicroBeads in PEB (Anti-SSEA-1 (CD15)-MicroBeads (Miltenyi Biotec 130-094-530), Anti-Biotin-MicroBeads (Miltenyi Biotec 130-090-485) and Anti-EpCAM-MicroBeads, respectively). Cell suspensions were then washed in PEB and resuspended in 0.5 ml iPS-Medium without LIF. Columns were pre-equilibrated and placed in appropriate magnets. Cells suspensions were administered and columns afterwards washed with medium by gravity flow. The entire flow-through was collected as negative fraction. Positive fractions were eluted in required volumes using the provided plunger. EPCAM and SSEA1 separations were carried out using MS columns, FAS separations using LD columns. After separation, cells were investigated by flow cytometry or seeded on top of CellTrace Violet Dye pre-stained (Invitrogen) gamma-irradiated feeder cells (1×10^5^ cells per well of a 12well plate). Seeded cells were further cultured in “3i” conditions for 6 days with media changes every other day.

### Establishment of iPSC lines after magnetic separation

Limiting dilutions were performed to isolate single cells after separation. Separation based on SSEA1 was performed at day 18 p.t. and the SSEA1 positive fraction was diluted in “3i” conditions to a cell density of 5 cells/ml. 0.1 ml of this cell suspension was seeded per well of a 96well plate containing gamma-irradiated CF1-MEFs and further cultured for 6–8 days. Single colonies were expanded and analyzed by flow cytometry. Lines that expressed highest levels of SSEA1 were subcloned by a second round of limiting dilutions to ensure clonality of derived iPSC lines.

### Teratoma assay

iPSC were harvested, 1×10^6^ cells resuspended in 0.2 ml PBS and injected subcutaneously into the flanks of six NOD.Cg-Rag1tm1Mom Il2tm1Wjl/Sz (NRG) mice. Teratoma formation occurred 4–8 weeks after injection. During this time frequent checking of the teratoma formation ensured the termination of the experiment at the approved state. The guidelines issued from the GV-Solas (Society for Laboratory Animal Science) and TVT (Veterinary association for Animal Welfare, Germany) served as basis for defining the humane endpoints. After anesthesia using carbon dioxide, animals were sacrificed by cervical dislocation. Teratomas were fixed in 4% neutral buffered formalin (pH 7.2), embedded in paraffin and 2 µm sections Hematoxylin & Eosin stained.

### Microarray meta-analysis

Publicly available microarray datasets were analyzed using the Genevestigator anatomy tool [28]. This tool displays expression levels of genes of interest in various tissues and cell lines. The expression level within a tissue type is the average expression across all samples that were annotated with that particular cell type. Data sets analyzed in this study were derived on Affymetrix microarray “Human133_2: Human Genome 47k array”.

### Statistical Analysis

Throughout the paper, p values were calculated with Student's t tests. *p<0.05, **p<0.01, ***p<0.001. Scale bars show the SD of at least three separate experiments unless otherwise stated (alpha = 95%).

## Supporting Information

Figure S1 **Expression intensity of candidate markers on the Oct4-GFP transgenic MEFs.** A) Mean fluorescence intensity of potential MEF associated markers as determined by flow cytometry. B) Expression levels of potential pluripotency associated markers.
(TIF)

Figure S2 **Expression characteristics of candidate markers on the different reprogramming subpopulations.** The expression frequencies of potential pluripotency associated markers (left column) and potential MEF associated markers (right column) as observed in cell subpopulations progressing through reprogramming. Frequencies were examined by flow cytometry (n = 3: mean +/− SD).
(TIF)

Figure S3 **Efficiencies of magnetic separations from reprogramming MEFs.** The frequencies of the respective markers used for separation are shown for unseparated fractions and target fractions (SSEA1^+^, EpCAM^+^ and FAS^−^).
(TIF)

Table S1 **Marker expression frequencies as detected in the antibody screening experiment.** Altogether 170 antibodies were screened. Given are details on antibody vendors and staining conditions. The frequencies of positive cells among CF1-MEFs, HM1-ESCs and LV1-7b-iPSCs (in percent) are listed for each individual replicate. n.a.  =  not available.
(XLS)

Checklist S1 (PDF)
