# Supplementary figures and images for: FAS-Based Cell Depletion Facilitates the Selective Isolation of Mouse Induced Pluripotent Stem Cells
Source: PLoS One. 2014 Jul 16;9(7):e102171. doi: 10.1371/journal.pone.0102171 (PMC4100888; doi:10.1371/journal.pone.0102171)

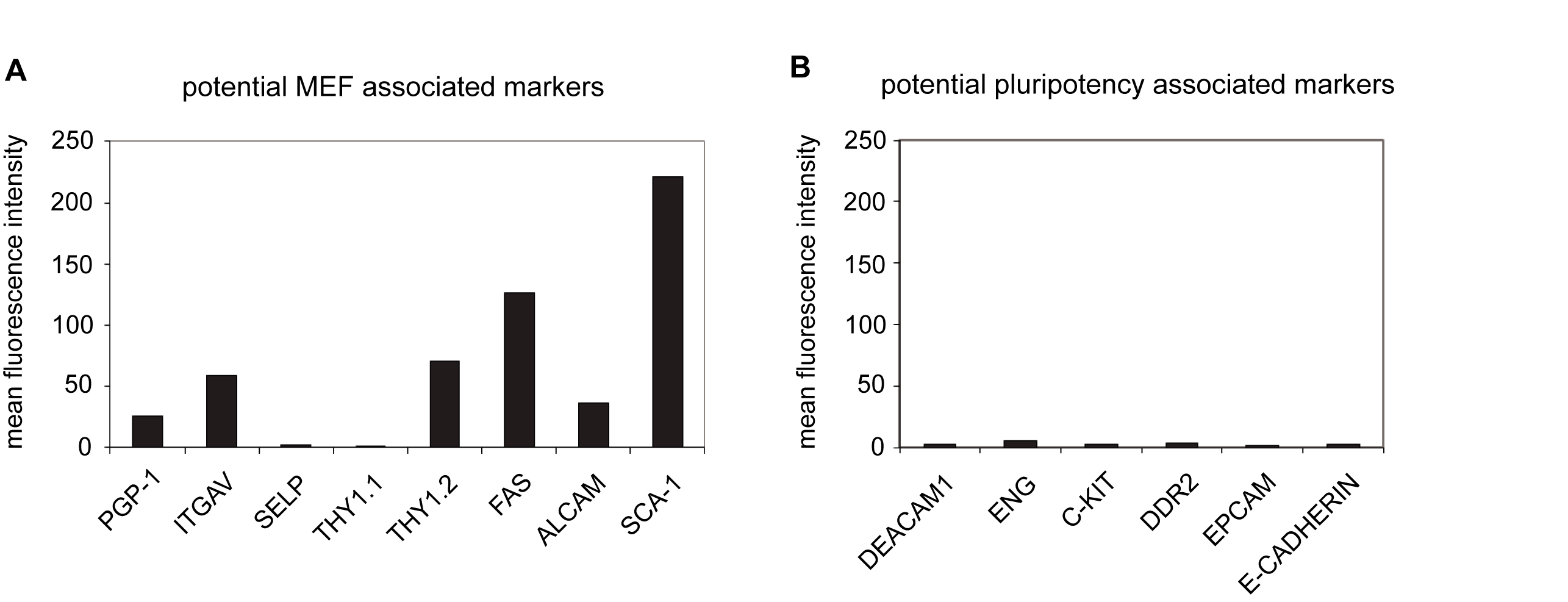

Supplement: Figure S1 — Expression intensity of candidate markers on the Oct4-GFP transgenic MEFs. A) Mean fluorescence intensity of potential MEF associated markers as determined by flow cytometry. B) Expression levels of potential pluripotency associated markers. (TIF) [file pone.0102171.s001.tif]

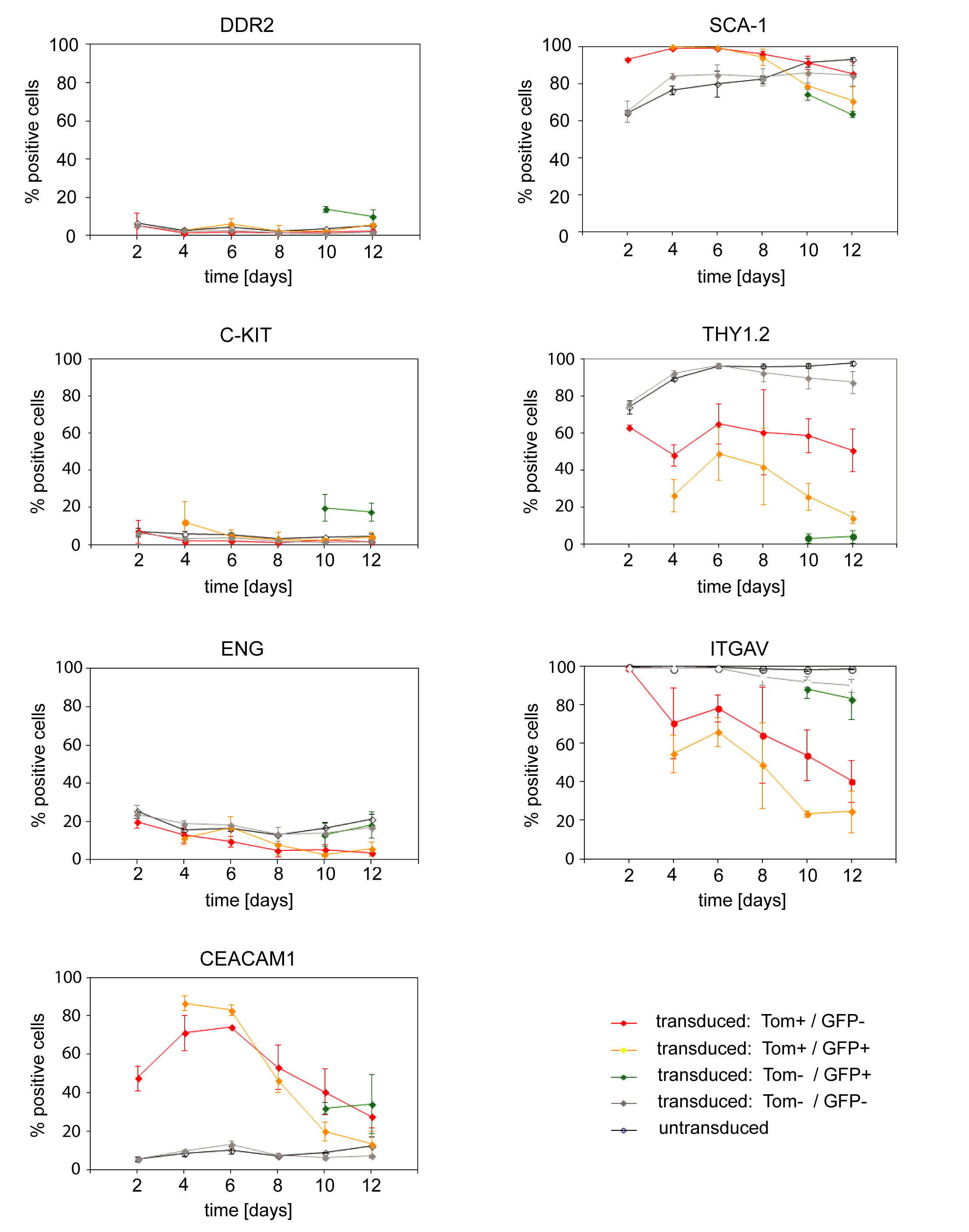

Supplement: Figure S2 — Expression characteristics of candidate markers on the different reprogramming subpopulations. The expression frequencies of potential pluripotency associated markers (left column) and potential MEF associated markers (right column) as observed in cell subpopulations progressing through reprogramming. Frequencies were examined by flow cytometry (n = 3: mean +/− SD). (TIF) [file pone.0102171.s002.tif]

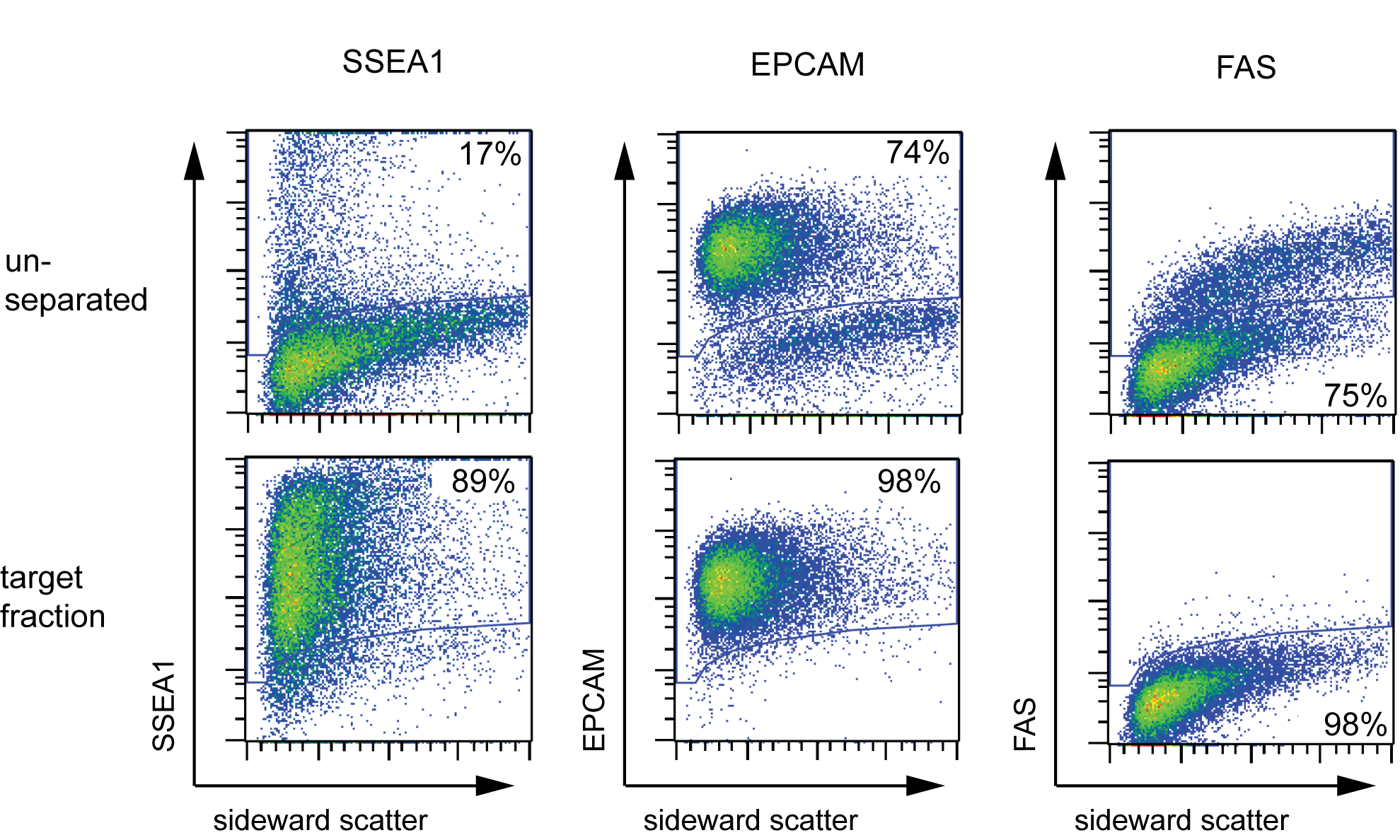

Supplement: Figure S3 — Efficiencies of magnetic separations from reprogramming MEFs. The frequencies of the respective markers used for separation are shown for unseparated fractions and target fractions (SSEA1+, EpCAM+ and FAS−). (TIF) [file pone.0102171.s003.tif]
